# 1793. Pipes as a Harm Reduction Tool: Insights from People Who Use Methamphetamine in Rural Illinois

**DOI:** 10.1093/ofid/ofad500.1622

**Published:** 2023-11-27

**Authors:** Alex Rains, Erin Augustine, John Bresett, Scott Fletcher, Kyle Miller, Rebecca Bolinski, Wiley D Jenkins, Mai T Pho, Jerel Ezell

**Affiliations:** University of Chicago, Chicago, Illinois; University of Chicago, Chicago, Illinois; Southern Illinois University, Carbondale, Illinois; Community Action Place, Murphysboro, IL; Southern Illinois University, Carbondale, Illinois; Southern Illinois University, Carbondale, Illinois; Southern Illinois University School of Medicine, Springfield, IL; University of Chicago Medicine, Chicago, IL; Weill Cornell Medicine, New York, New York

## Abstract

**Background:**

Methamphetamine (meth) use is disproportionately high in rural settings and increased during COVID-19. While sterile syringes services are an evidence-based measure to reduce infectious disease transmission, little research exists around the value of smoking equipment, specifically pipes, in minimizing harms associated with rural drug use in the US.

**Methods:**

Between March and June 2022, we conducted structured interviews with people who use methamphetamine (PWUM) living in rural Illinois who were at least 15 years old and used meth by any route in the past 30 days. Interview guides explored attitudes and behaviors regarding meth use routes, meth pipe use practice, and pipe access, and were refined by a peer-led Community Advisory Board. Interviews were audio recorded, transcribed, and coded in MAXQDA. The data were then analyzed for emergent themes using modified grounded theory.

**Results:**

Nineteen participants, average age 37.1 (SD+8.7), were interviewed. Of these, 53% were women, and 89% were white. All reported smoking meth, and 84% injected meth. Participants reported that they often chose to smoke rather than inject to mitigate health concerns like wounds, pain, and infections. Smoking enabled them to use around others as opposed to using alone (as was typical when they injected). Participants expressed interest in pipe distribution through a harm reduction agency, e.g. according to one participant (a 31-year-old white male), “I’ve asked specifically if they had a pipe because I was trying to lay off the syringes.” They related that access to a harm reduction agency distributing pipes would connect people to other services such as HIV testing, naloxone, and safer sex supplies.

**Conclusion:**

Distribution of meth pipes through harm reduction agencies represents a means for decreasing harm through circumvention of injection, decreased solitary drug use, and linkage to additional services related to overdose and disease prevention. Given increased rates of meth use in rural regions, this intervention could specifically address drug-related harms that impact rural PWUM. Future work should quantitatively explore the health impacts of pipe use as a harm reduction intervention.

**Disclosures:**

**All Authors**: No reported disclosures

